# Messenger RNA-encoded reporters for monitoring cellular stress and bioenergetics

**DOI:** 10.1038/s41598-026-49851-y

**Published:** 2026-06-17

**Authors:** Karen Kai-Lin Hwang, Qi Fang, Carine Nizard, Anne-Laure Bulteau, Knut Woltjen

**Affiliations:** 1 Department of Life Science Frontiers, Center for iPS Cell Research and Application (CiRA), Kyoto University, 53 Kawahara-cho, Shogoin, Sakyo-ku, Kyoto, 606-8507 Japan; 2 Graduate School of Medicine, Kyoto University, Kyoto, 606-8507 Japan; 3 Life Science Department, LVMH Recherche, 45800 Paris, France

**Keywords:** Metabolic reporters, Bioenergetics, pH, ATP, Reactive oxygen species, Messenger RNA, Biological techniques, Biotechnology, Cell biology, Stem cells

## Abstract

The ability to monitor cellular processes in real-time is essential for understanding cell function, disease progression, and therapeutic responses. Engineered reporter proteins have been developed for monitoring cellular metabolism, stress responses, and bioenergetics. However, their use in primary cells is limited by inefficient plasmid transfection and the impracticality of generating and validating stable cell lines for each application. Here, we use in vitro transcription to generate mRNA-encoded metabolic trackers and achieve high transfection efficiencies in primary fibroblasts, cancer cells, and induced pluripotent stem cells. This approach provides a flexible platform for real-time monitoring of cellular processes in diverse cell types and overcomes the technical barriers of establishing stable cell lines by genetic modification. We confirm the activity of three ratiometric reporters that monitor pH, H_2_O_2_, and ATP levels in subcellular compartments. Our mRNA-based approach provides a versatile, efficient tool for real-time metabolic studies across basic and applied research, reducing reliance on commercially available reporters and broadening the applicability of metabolic reporters in patient-derived cell models.

**Supplementary Information:**

The online version contains supplementary material available at 10.1038/s41598-026-49851-y.

## Introduction

Studies of cellular metabolism, stress responses, and bioenergetics often rely on engineered reporter proteins to detect intracellular dynamics. However, the establishment of primary cell lines expressing reporter proteins is a significant technical limitation. The use of messenger RNA (mRNA) to transiently deliver genetic information has substantially changed the biomedical field, especially following the development of COVID-19 mRNA vaccines^1,2^. The efficacy and safety of mRNA in both mitotic and post-mitotic cells arise from its high transfection efficiency, ability to rapidly translate into functional proteins within the cytoplasm, and lack of genome integration^3,4^. Conventional plasmid DNA-based delivery, while widely adopted in molecular biology studies, often suffers from poor transfection efficiency and slow response time, especially in challenging-to-transfect cell types such as primary fibroblasts and pluripotent stem cells (PSCs), due to the extra need for nuclear entry and transcription^5^. While establishing stable cell lines remains a strategy for introducing exogenous genes in PSCs, the limited proliferative capacity of primary cell lines makes it impractical for such applications^6^. As a result, reporter-expressing primary cell lines are rarely adopted in the field.

In comparison to plasmid or transgene delivery, mRNA-based methods generally allow for higher and more rapid protein production^7,8^. Previous studies have introduced the use of mRNAs for applications such as organelle labelling and luciferase assays^9,10^. Despite this, the perceived instability of mRNA has historically limited its adoption in molecular biology^11^. This fragility, often attributed to degradation at room temperature and sensitivity to freeze-thaw cycles, contrasts with the need for robust, reproducible tools in experimental workflows^12,13^.

Here, we present a method for leveraging mRNA to deliver reporter genes with high efficiency and adaptability across diverse cell types, including primary fibroblasts, cancer cells, and induced pluripotent stem cells (iPSCs). Our approach builds on prior mRNA-based strategies by optimizing in vitro transcription (IVT) for stable and easy-to-produce mRNA encoding fluorescent reporters of cellular metabolism with customizable subcellular localization signals. We demonstrate the high specificity and design flexibility of this method, along with the stability of IVT-synthesized mRNAs. Our study highlights mRNA as a versatile platform for monitoring subcellular environments under cell physiological conditions, offering a powerful and economical alternative for in vitro metabolic studies using primary cells.

## Results

### mRNA-encoded fluorescence reporters for effective labeling of subcellular structures

**Fig. 1 Subcellular localization of mRNA-encoded reporters:**
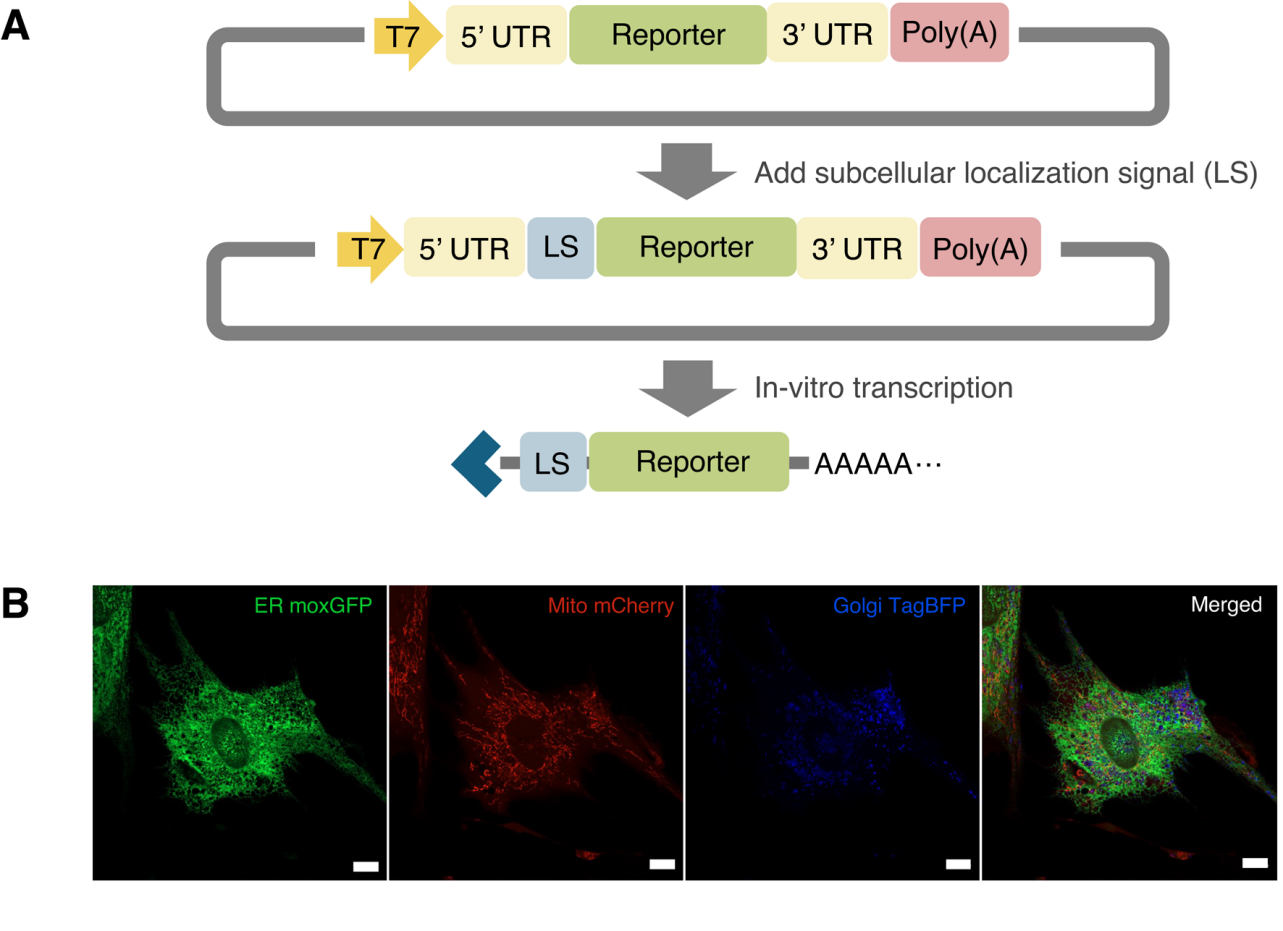
**A)** Schematic of in vitro transcription (IVT) template design for subcellular targeting. Localization signals were added to the N-terminus of reporter proteins for subcellular targeting. T7 – T7 polymerase promoter; 5′/3′UTR, untranslated region; LS, localization signal. The template is used for IVT to produce reporter mRNA. **B)** Pseudo-coloured confocal microscopy images of mRNA-encoded fluorescent proteins targeting the endoplasmic reticulum (ER) (green), mitochondria (red), and Golgi apparatus (blue). Scale bars indicate 10 μm.

Fluorescence reporter mRNAs are commonly used as positive controls for transfection experiments. Compared to plasmid DNA transfection, mRNA delivery provides accelerated transient expression and high transfection efficiency^7,14^. While mRNA transfection is advantageous for many applications, a high level, acute expression can overwhelm the cell’s intrinsic protein folding, modification, and trafficking machinery, thereby posing challenges for the accurate and efficient subcellular localization of proteins bearing specific targeting signals^15^. To assess the specificity of localization, we generated mRNA-mediated reporters for subcellular compartments, where a subcellular localization signal (LS) is added in addition to reporter proteins in the IVT template vector (Fig. 1A). We generated mRNA for fluorescent proteins including mitochondria-targeted mCherry^16^, ER-targeted moxGFP^17^, and Golgi-targeted TagBFP^18^, then transfected these mRNAs into U2OS cells to validate the specificity of localization signals. At 24 h post-transfection, we observed distinct fluorescence from each reporter protein, demonstrating the high localization specificity of these proteins (Fig. 1B, S1).

### mRNA-encoded reporters for mitophagy and ATP production

**Fig. 2 Detection of mitophagy using an mRNA-encoded reporter.**
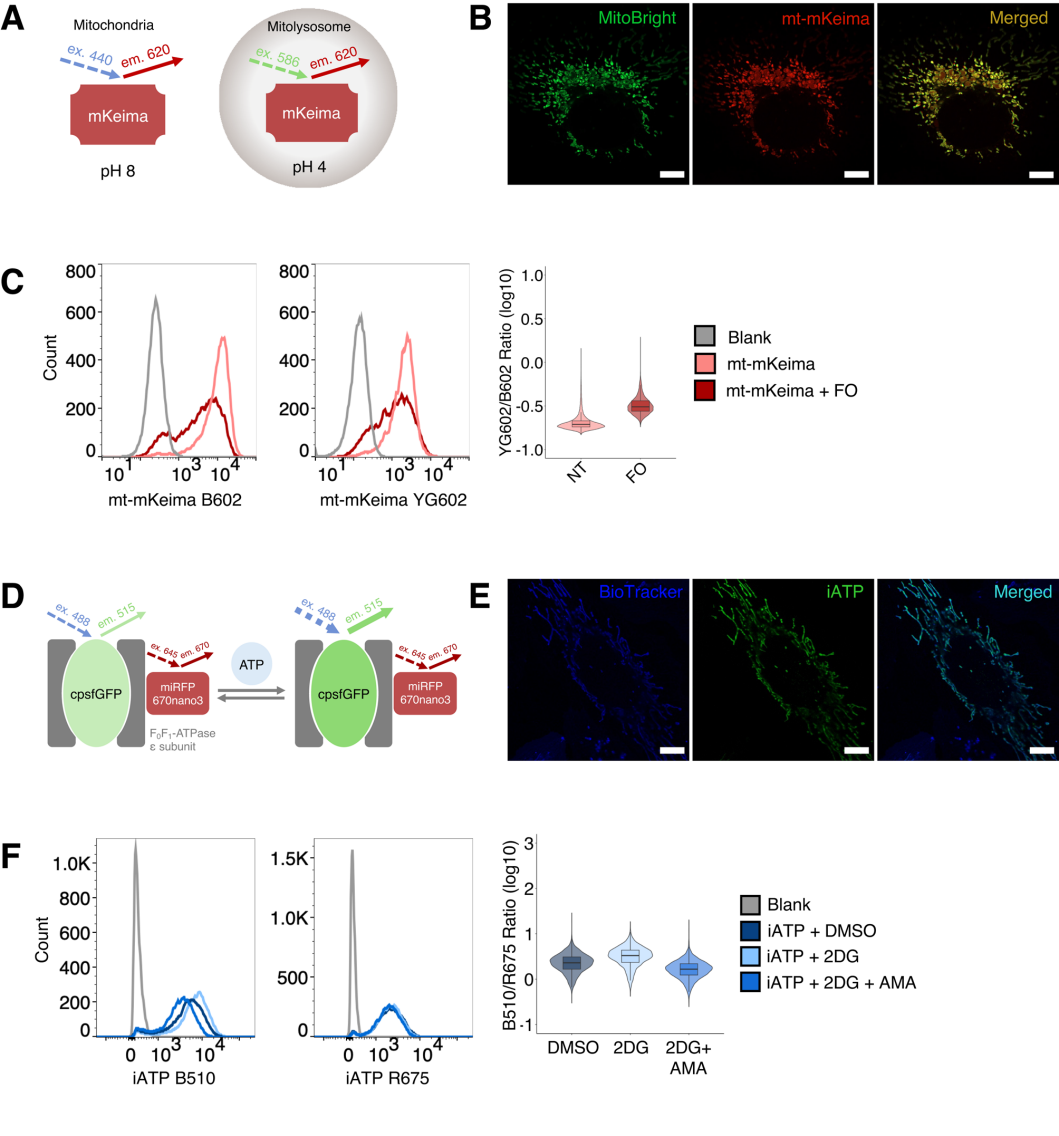
**A)** mKeima mechanism of action. At pH 8 in the mitochondrial matrix, mKeima has an excitation peak of 440 nm and an emission peak of 620 nm. When mitochondria fuse with lysosomes at pH 4, the excitation peak shifts to 586 nm. **B)** Pseudo-coloured confocal microscopy images of mitochondria-localized mKeima co-expressed with the mitochondria dye MitoBright LT Green in U2OS cells. mKeima (red) was specifically targeted to mitochondria using the COX8 mitochondrial localization signal. Scale bars indicate 10 μm. **C)** Flow cytometry assessment of mKeima response to mitophagy inducers FCCP and oligomycin A (FO) in BJ fibroblasts. More than 10,000 singlet events were collected for each analysis. **D)** iATP mechanism of action. iATP consists of a circularly permuted superfolder green fluorescent protein (cpsfGFP) embedded between the ATP-binding domains of the ε-subunit of a bacterial F_0_-F_1_ ATPase, which increases in fluorescence upon ATP binding. The construct is fused to a mitochondria localization signal and a constitutively expressed far red fluorophore (miRFP670nano3), which allows it to function as a ratiometric ATP sensor. **E)** Pseudo-coloured confocal microscopy images of mitochondria-localized iATP co-expressed with the BioTracker 405 Blue Mitochondria Dye in U2OS cells. iATP was specifically targeted to mitochondria using the COX8 mitochondrial localization signal. Scale bars indicate 10 μm. **F)** Flow cytometry assessment of iATP response to 2-deoxy-D-glucose (2DG) and antimycin A (AMA) in BJ fibroblasts. iATP was conjugated with miRFP670nano3 and tagged with a 4× COX8 mitochondrial localization sequence for targeted mitochondrial localization. More than 10,000 singlet events were collected for each analysis.

After observing the high specificity of mRNA-encoded fluorescent proteins, we decided to extend the application to metabolic reporter proteins. For this purpose, we produced mRNA for the previously published mitophagy reporter mt-mKeima^19^. mKeima is a fluorescent protein that has a pH-dependent excitation spectrum and constant emission spectrum (Fig. 2A). When fused to a mitochondrial localization signal (mt-mKeima), it can be used as a mitophagy tracker, as the excitation spectrum shifts during mitophagy when mitochondria are exposed to the acidic environment of the lysosome to form a mitolysosome^19^. The mitochondrial matrix has a resting pH of ~ 8. At this state, mKeima has an excitation peak of 440 nm and an emission peak of 620 nm. However, the excitation peak shifts to 586 nm at the lysosomal pH of ~ 4 (Fig. 2A). We first tested the localization of the mt-mKeima. After transfecting the mt-mKeima mRNA into U2OS cells, we stained the cells with the mitochondrial dye MitoBright LT Green and observed clear, overlapping signals, indicating that the mRNA-encoded mt-mKeima can successfully localize to the mitochondria (Fig. 2B). To validate reporter function in primary cells, BJ fibroblasts were first transfected with mt-mKeima mRNA, then treated 24 h later with 5 µM of FCCP and Oligomycin (FO) for 24 h to promote mitophagy by disrupting the mitochondrial membrane potential. mt-mKeima-transfected cells treated with FO showed an increase in the YG602/B602 ratio (Fig. 2C), suggesting the increased acidity experienced by the mitochondria as they fuse with lysosomes. These results show that mRNA-encoded mt-mKeima is functional as a mitophagy reporter.

To capture an additional dimension of cellular metabolism, we decided to assess the functional response of the ATP reporter iATPSnFR2 (hereafter referred to as iATP)^20,21^. iATP consists of a circularly permuted superfolder green fluorescent protein (cpsfGFP) embedded between the ATP-binding domains of the ε-subunit of a bacterial F_0_–F_1_ ATPase, which increases in fluorescence upon ATP binding (Fig. 2D)^21^. The construct is fused to a mitochondrial localization signal and a constitutively expressed far red fluorophore (miRFP670nano3), which allows us to track mitochondrial ATP via the green to far red ratio (B510/R675)^20^. Similar to mt-mKeima, the localization signal allows iATP to target the mitochondria, as demonstrated by its colocalization with the BioTracker 405 Blue Mitochondria Dye (Fig. 2E). To assess iATP response, BJ fibroblast cells were transfected with iATP mRNA for 24 h, then treated with 5 mM of the glycolysis inhibitor 2DG for another 24 h. Treatment of 2DG blocks glycolysis, thus promoting oxidative phosphorylation and mitochondrial ATP production^22^. Six hours prior to the flow cytometry analysis, DMSO or 10 mM Antimycin A was added to the cells. We observed that the R675 signals stayed consistent in all conditions (Fig. 2F). B510/R675 increased with the 2DG treatment, indicating an increase in mitochondrial ATP synthesis after glycolysis was inhibited (Fig. 2F). The addition of Antimycin A, which is a known inhibitor of complex III in the mitochondrial electron transport chain, abolished mitochondrial ATP synthesis, decreasing B510/R675(Fig. 2F). These results indicate that iATP, when delivered as mRNA, maintains its responsiveness as an ATP reporter in mitochondria.

### An mRNA-encoded reporter for oxidative stress

**Fig. 3 Detection of reactive oxygen species in the cytoplasm and mitochondrial space.**
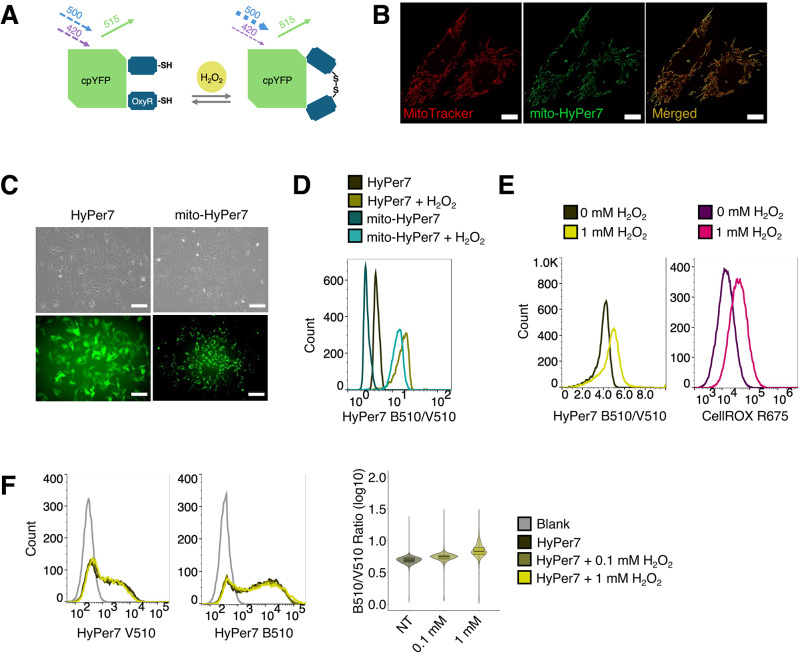
**A)** HyPer7 mechanism of action. HyPer7 contains a circularly permuted YFP (cpYFP) embedded in the regulatory domain of OxyR, an H_2_O_2_-sensing domain of *E. coli.* At basal levels, HyPer7 has two excitation peaks with maxima at 420 nm and 500 nm, and one emission peak at 516 nm. Upon H_2_O_2_ binding, the 420 nm excitation peak decreases proportionally to the increase in the 500 nm peak. **B)** Pseudo-coloured confocal microscopy images of mitochondria-localized HyPer7 co-expressed with MitoTracker Deep Red FM in U2OS cells. HyPer7 was specifically targeted to mitochondria using the COX8 mitochondrial localization signal. Scale bars indicate 10 μm. **C)** Fluorescence microscopy images of HyPer7 and mito-HyPer7 under basal conditions. U2OS cells were transfected with 250 ng of HyPer7 or mito-HyPer7 mRNA. Images were acquired using a GFP filter 24 h post-transfection. **D)** Flow cytometry assessment of the H_2_O_2_ response of HyPer7 and mito-HyPer7 in U2OS cells. An oxidizing environment was induced by 200 µM H_2_O_2 _ for 30 min. More than 10,000 singlet events were collected for each analysis. **E)** Comparison of H_2_O_2_ dose response between HyPer7 and commercial ROS dye. U2OS cells were transfected with HyPer7 mRNA or stained with the commercial ROS sensor CellROX, and treated with 0 mM, 1 mM, or 3 mM of H_2_O_2_. More than 10,000 singlet events were collected for each analysis. **F)** Flow cytometry assessment of the response of HyPer7 to different doses of H_2_O_2_ in BJ cells. The HyPer7 ratio (B510/V510) increases with the H_2_O_2_ concentration. More than 10,000 singlet events were collected for each analysis.

We next expanded the use of metabolic tracker mRNA to assess cellular oxidative stress. HyPer7, a ratiometric fluorescent reporter for intracellular H_2_O_2_, consists of a circularly permuted YFP (cpYFP) embedded in the regulatory domain of OxyR, an H_2_O_2_-sensing domain of *E. coli*^23^. At basal levels, HyPer7 has two excitation peaks with maxima at 420 nm and 500 nm, and one emission peak at 516 nm (Fig. 3A). Upon H_2_O_2_ stimulation, the 420 nm excitation peak decreases proportionally to the increase in the 500 nm peak (Fig. 3A). Like the previous two metabolic reporters, the addition of a mitochondrial localization signal to HyPer7 (mito-HyPer7) allows it to colocalize with MitoTracker (Fig. 3B). The difference in fluorescence distribution between unlocalized HyPer7 and mito-HyPer7 allows differing levels of H_2_O_2_ to be captured (Fig. 3C). In fact, different B510/V510 ratios were observed between HyPer7 and mito-HyPer7, capturing the distinct redox environments in the two subcellular fractions (Fig. 3D). In U2OS cells, upon exposure to 200 µM of H_2_O_2_, a significant increase in the B510/V510 ratio was observed in both reporters, confirming that the reporter function is maintained in both cytoplasmic and mitochondrial environments (Fig. 3D). We then compared the mRNA-delivered HyPer7 to CellROX, a commercially available dye-based alternative for sensing oxidative stress. Both HyPer7 and CellROX showed an increase in response that positively correlates with the H_2_O_2_ concentration (Fig. 3E). This data indicates that the behavior of mRNA-encoded HyPer7 performs comparably to commercially available chemical sensors. To assess the dose response of HyPer7, BJ fibroblasts were transfected with the reporter mRNA one day before H_2_O_2_ treatment (0, 0.1, and 1 mM) using flow cytometry. We successfully observed that the B510/V510 ratio increased with the H_2_O_2_ concentration (Fig. 3F).

With the functionality and localization of the HyPer7 reporter confirmed, we next evaluated its expression kinetics and stability under practical conditions. The reporter showed rapid expression, with the HyPer7 signal detected just 2 h after transfection in U2OS cells (Fig. S2A). By 4 h, the reporter had already developed a robust response to H_2_O_2_ (Fig. S2A). In addition to the efficacy and robustness, we also explored whether the mRNA-encoded reporters would be stable enough for regular use. Specifically, we mimicked the practical storage and usage scenario and subjected the HyPer7 mRNA to multiple freeze-thaw cycles. We then performed transfection to validate the freeze-thawed mRNAs using flow cytometry. Flow cytometry results showed no visible differences in reporter activity even after 10 freeze-thaw cycles, suggesting the mRNA maintained its function (Fig. S2B). TapeStation analysis of mRNA integrity further confirmed that the transcripts remained intact following freeze-thaw cycles (Fig. S2C). Overall, the ability for mRNA-encoded reporters to maintain structural integrity and robust expression in practical use scenarios allows them to be applied to a wide range of assays.

### Application of the mRNA-encoded ATP synthesis reporter in MELAS iPSCs

**Fig. 4 Assessment of mitochondrial ATP levels in iPSCs from patients with metabolic disease.**
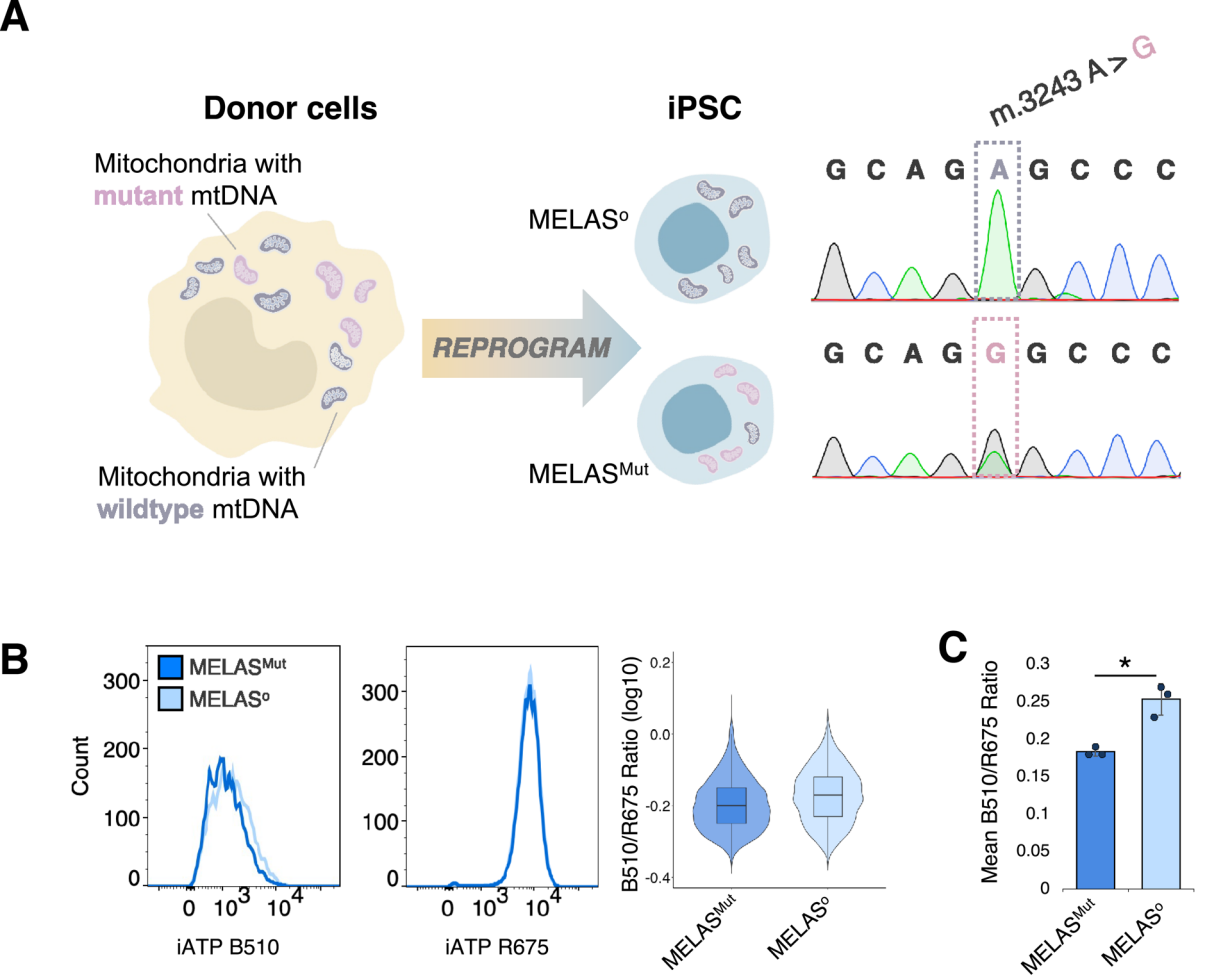
**A)** MELAS clones from the same donor have varying levels of m.A3243G mutation load. Clone MELAS^o^ has a m.A3243G mutation load of 0% and Clone MELAS^Mut^ has a m.A3243G mutation load of 67%. **B)** Flow cytometry assessment of iATP response in MELAS^Mut^ and MELAS^o^ cells. MELAS patient-derived iPS cells were transfected with 250 ng of iATP mRNA. More than 10,000 singlet events were collected for each analysis. **C)** Mean iATP ratio (B510/R675) of MELAS^Mut^ and MELAS^o^ cells (*n* = 3). Statistical significance was assessed using a two-tailed Student’s t-test; *p* < 0.05 is denoted by an asterisk. Error bars represent standard deviation.

Finally, we applied the iATP mRNA in iPSCs derived from a patient with mitochondrial encephalomyopathy, lactic acidosis, and stroke-like episodes (MELAS)^24^. MELAS syndrome, caused by the m.3243 A > G mutation in mitochondrial DNA, impairs mitochondrial function and decreases ATP production, allowing it to serve as a useful system for validating mRNA-based metabolic reporters like iATP^25,26^. For this purpose, we selected two iPSC clones from a MELAS patient^24^: MELAS^o^ with no detectable mutant load, and MELAS^Mut^ harboring ~ 67% mutant load (Fig. 4A). After transfecting the MELAS^o^ and MELAS^Mut^ cells with 250 ng of iATP mRNA, we plotted the changes in fluorescent intensity for each channel and calculated the B510/R675 ratios (Fig. 4B). As expected, the MELAS^Mut^ clone exhibited a lower B510/R675 ratio compared to the MELAS^o^ clone (Fig. 4C). Particularly, the mean B510/R675 ratio is approximately 30% lower in the MELAS^Mut^ clone (Fig. 4C). Therefore, mRNA-encoded iATP can serve as a rapid and efficient system to compare intracellular ATP dynamics in iPSCs.

## Discussion

In this study, we demonstrated the potential of using mRNA as a delivery platform for reporter proteins to investigate metabolic dynamics in various cell types. We applied this system in MELAS patient-derived iPSCs and successfully proved its efficacy. While these novel tools have been proven to work in stable cell lines, we demonstrated that these reporters can be efficiently delivered as mRNAs. Traditionally, methods such as the Seahorse assay are used to monitor metabolic status^27,28^. However, for cells like iPSCs that have unique metabolic profiles and growth characteristics, the assay requires careful optimization^29^. Other methods, like the ATP bioluminescence kit, are designed for testing whole cells rather than specific organelles^27^. Moreover, these alternative methods cause cells to be unusable after measurement, making it difficult for longitudinal studies such as those assessing cell recovery to stress^27,28^. Similarly, while oxidative stress production can be measured by CellROX or related chemical sensors, their irreversible nature limits the readouts to a cumulative measurement^30^. Since mRNA transfections maintain the biological integrity and spatial information of the sample, mRNA may be useful for longer term experiments that involve serial treatments. Notably, this approach allows for the implementation of reporters like iATP, for which no equivalent commercial dyes are currently available. Furthermore, chemical sensors for oxidative stress are oxidized by several sources of ROS^30^, making the readouts less specific than HyPer7, which directly reacts with H_2_O_2_^23^. Dyes like JC-1, which measures mitochondrial membrane potential, or MitoTracker, which measures mitochondrial mass, are sometimes used as indirect mitophagy reporters^31^. In comparison, mt-mKeima quantifies the mitophagic flux, providing a direct measure of the degradation process^19^. The ability of mRNA reporters to capture fluctuating metabolic states longitudinally serves as an effective alternative to monitor intracellular dynamics in real-time. Additionally, multiple reporter mRNAs can be synthesized in parallel on the same day, with enough yield to last for multiple assays across different cell lines. This approach not only requires less time and effort compared to establishing a stable reporter-expressing cell line but also offers the option to use multiple reporters simultaneously, allowing for the measurement of different metabolic processes in several subcellular compartments. Moreover, both our experience and previous reports have indicated that mRNA can achieve higher transfection efficiency while retaining the flexibility of plasmid DNA transfection, making it applicable to a wider range of cell types^7,14^. Additionally, mRNA avoids the risk of unwanted genome integration posed by DNA-based methods, which helps preserve the native cell function after an experiment concludes^3,4^. While mRNA is known to degrade easier compared to DNA^11^, our results also challenge the impression of mRNA’s fragility. When handled appropriately, IVT-synthesized mRNAs exhibit stability at the molecular and functional levels^32^. This finding aligns with emerging evidence that capping analogs, such as CleanCap^®^, may enhance mRNA stability, broadening its practical utility^1^.

Collectively, these advantages effectively reduce assay turnover time and position the use of mRNA as a more suitable and feasible tool for the study of primary cells with finite lifespans. In comparison to traditional viral-mediated or transposase-mediated integration^33^, which often requires additional steps of selection and expansion, using mRNA-based reporters can bypass the replicative senescence and potential changes to the metabolic profile during the establishment of reporter cell lines^34^. This can significantly impact studies of mitochondrial diseases. For example, MELAS patient-derived iPSCs are more sensitive to selection drugs such as puromycin, which has been used as a mitochondrial translation inhibitor^35,36^. With our mRNA-based reporter system, we could minimize the potential selection-induced population bias caused by the level of mutant heteroplasmy.

Nevertheless, certain limitations remain. While both plasmid-based DNA-based transfection approaches and plasmid-based IVT templates for mRNA require cloning, bacterial amplification, and DNA purification, the additional steps of construct linearization and IVT are needed to produce mRNA. Similar to plasmid transfection-based methods, mRNA-encoded fluorescent protein reporters remain transiently expressed in the cells. A primary downside of transient transfections is the susceptibility to heterogeneous uptake. This issue can be alleviated by using dosage-independent mRNA reporters. In contrast, stable cell lines generally offer more uniform and longer-lasting expression for more consistent results^37^. While mRNA bypasses the need for nuclear entry and provides an accelerated response compared to plasmid transfection^7,14,38^, the need for active translation still introduces a temporal delay in comparison to protein-based reagents^39^. Similar to DNA replication, protein translation is a highly energy-consuming event in cells. As such, the mRNA amount must be carefully adjusted to avoid overwhelming the translation machinery and altering the metabolic dynamics. In fact, cellular resources need to be reallocated for cells to translate reporter mRNAs, which could lead to changes in cellular behavior associated with metabolic burden^40^. It is known that in bacteria and single-cell eukaryotes, protein overexpression can cause the formation of inclusion bodies with misfolded proteins, triggering multiple secondary cytotoxic effects^41–43^. Since mRNA is the direct medium for nascent protein synthesis, when a high copy of reporter coding mRNA is delivered to cells, it is possible to cause misfolding and artificial secondary effects. Therefore, dosage optimization is critical when using mRNA-delivered reporters. Finally, the choice of transfection reagents and variations in efficacies across cell lines should also be taken into consideration.

Promising areas for future development include mRNA-encoded modifications that enhance the stability and specificity of metabolic reporter expression. Stability can be increased with modifications to RNA structure. For example, using structures such as circular RNAs (circRNA) could prevent degradation by exonucleases^44^, while incorporating self-amplifying RNA (saRNA) architectures can allow the RNA to replicate and persist in cells for longer^45^. In addition, incorporating miRNA-based incoherent feed-forward loops (iFFLs) into the template design could dynamically mitigate the metabolic burden and stabilize reporter expression^40^. Beyond stabilization, feed-forward loops can take metabolic stress signals as inputs for therapeutic RNA circuits, where a signal intensity above a certain threshold triggers the translation of a downstream product to combat the stress. Furthermore, incorporating technologies such as a split RNA switch can vastly improve specificity, allowing mRNA-encoded reporters to track specific cell types in a heterogeneous population^46^. The versatility of RNA regulation systems allows for a growing variety of customizations that can expand the utility of mRNA-encoded reporters.

## Conclusion

In conclusion, we have verified the efficacy of mRNA-delivered fluorescent protein reporters across multiple cell lines, highlighting their advantages in transfection efficiency, flexibility, and stability. While some limitations persist, our study provides valuable evidence for mRNA as a versatile mode of metabolic reporter delivery. As new biosensors are actively being developed, the array of tools that can be applied to metabolic studies continues to expand^47,48^. Ultimately, this approach holds significant promise for studying subcellular environments, cellular stress, and bioenergetics in both basic research and applied settings.

## Methods

### Ethics approval

All experiments involving human cell lines were performed in accordance with relevant guidelines and regulations. Informed consent was obtained from all donors. The study was conducted following the ethical approval for human disease and gene analysis (R0091/G259, G0687) by the Kyoto University Graduate School and Faculty of Medicine, Ethics Committee.

### Cell lines and cell culture

201B7 (HPS0063)^49^, 409B2 (HPS0076)^50^, MELAS^Mut^ (HPS2866)^24^ and MELAS^o^ (HPS2869)^24^ human induced pluripotent stem cell lines are available from the RIKEN BioResource Research Center Cell Bank. U2OS cells (HTB-96) and BJ fibroblasts (CRL-2522) were obtained from ATCC. Human induced pluripotent stem cells were maintained at 37 °C in 5% CO_2_ and AK02N medium (Ajinomoto) on laminin-coated wells (iMatrix silk 511) as previously described^51^. U2OS cells and BJ fibroblasts were maintained at 37 °C in 5% CO_2_ and Dulbecco’s modified Eagle’s medium (Nacalai Tesque) supplemented with 10% FBS (Gibco) and 100 U/mL penicillin-streptomycin (Gibco).

### Plasmid construction

Sequences for fluorescent reporters and localization signals were obtained from Addgene (#131626 pHAGE-mt-mKeima, #68072 ERmoxGFP, #55102 mCherry-Mito7, #206259 Golgi-TagBFP) or synthesized by Twist Bioscience (mito-HyPer7 and iATP) and PCR amplified using the KOD One PCR Master Mix (Toyobo). PCR products were incubated at 37 °C for 1 h with DpnI (NEB) to remove template DNA, then heated at 70 °C for 1 min to inactivate DpnI. Fragment sizes were verified using gel electrophoresis, and the products were purified with the Monarch PCR & DNA Cleanup Kit (NEB). Primer design and In-Fusion procedures were performed according to the IVTpro (BspQI) mRNA Synthesis System (Takara).

The resulting In-Fusion products were transformed into Competent Quick DH5α (Toyobo). The transformation mixture was smeared onto LB agar plates with 50 µg/mL kanamycin and left to grow overnight at 37 °C. Single colonies were picked and expanded in LB medium with 50 µg/mL kanamycin for 16 h, then purified for plasmid DNA using the Wizard Plus SV Minipreps and DNA Purification System (Promega). Purified plasmids were verified using whole plasmid sequencing (Plasmid-EZ, Azenta Life Sciences). The IVT plasmid template was linearized using BspQI-HF (NEB) and purified using the Monarch PCR & DNA Cleanup Kit (NEB).

### In-vitro transcription

With slight modifications to the manufacturer’s protocol, the IVTpro (BspQI) mRNA Synthesis System (Takara) was used for the mRNA synthesis. Typically, 300 ~ 500 ng of linearized IVT template was used for mRNA synthesis, and CleanCap AG (TriLink) was used as a capping analog. The reaction was incubated at 37 °C for 4 h, subsequently treated with DNaseI (Takara) to remove the linearized template DNA. The resulting mRNA was then purified using the Monarch RNA Cleanup Kit (NEB). Purified mRNAs were treated with Antarctic phosphatase (NEB) to remove the 5′-triphosphate group, followed by another round of purification. mRNA concentration was measured using Nanodrop, and the quality was assessed by TapeStation 4200 (Agilent) using RNA ScreenTape following the manufacturer’s protocol (Agilent). The purified mRNAs were aliquoted into nuclease-free screw cap tubes (Axygen) and stored at − 80 °C at a concentration of 1 µg/mL.

### mRNA stability

mRNA freeze-thawing was performed to mimic daily use. Briefly, tubes containing mRNAs stored at − 80 °C were first thawed completely to room temperature, then placed on ice for 5 min before returning to − 80 °C. Every freeze-thaw cycle was performed with at least a 24-hour interval. mRNA quality was assessed using TapeStation 4200 (Agilent) at the end of the experiment.

### Transfection

For mRNA transfections in U2OS, BJ, and 409B2, and MELAS iPSCs, both forward and reverse transfections were performed using Lipofectamine MessengerMAX (Thermo Fisher Scientific) according to the manufacturer’s protocol. For reverse transfections in U2OS and BJ cells, Lipofectamine Stem (Thermo Fisher Scientific) was used for 409B2 cells. In brief, cells were harvested with TrypLE Express (Gibco) and counted using the Countess Automated Cell Counter. Cells were then resuspended in Opti-MEM (Gibco) and mixed with the mRNA/DNA complex before plating. 500ng of mRNA was used to transfect approximately 200,000 cells per well of a 12-well dish.

### Microscopy

Localization of ER moxGFP, Mitochondria mCherry, Golgi TagBFP, and mt-mKeima were examined using a Zeiss LSM900 with AiryScan2 at one day post-transfection. Before imaging the mt-mKeima condition, cells were stained with the mitochondria dye MitoBright LT Green (Dojindo), BioTracker 405 Blue Mitochondria Dye (Sigma-Aldrich), or MitoTracker Deep Red FM (according to the manufacturer’s protocol). HyPer7 and mito-HyPer7 localization were assessed by the Keyence BZ-X810 fluorescence microscope with a GFP filter at one day post-transfection.

### Flow cytometry

Flow cytometry was performed with the FACSymphony A5 SE Cell Analyzer (BD). Briefly, cells were harvested with TrypLE Express (Gibco) and neutralized with FACS buffer - FluoroBrite DMEM (Gibco) + 5% FBS (Gibco). Cells were then resuspended in FACS buffer and placed on ice before the analysis. For H_2_O_2_ treatment, H_2_O_2_ (Nacalai Tesque) was diluted in culture media and incubated for 30 min before harvest. For FCCP and Oligomycin A treatment, 5 µM FCCP and Oligomycin A (Abcam) were added to the cells transfected with mt-mKeima and incubated overnight at 37 °C in 5% CO_2_. For 2DG and Antimycin A treatment, 5mM 2DG (Nacalai Tesque) or 2mM 2DG + 10mM Antimycin A (Sigma) was added to the cell transfected with iATP and incubated overnight at 37 °C in 5% CO_2_ with media supplied with additional 1mM sodium pyruvate (Nacalai Tesque). For the time-course experiment, U2OS cells were first seeded onto a 6-well plate, then the cells were transfected with 500 ng HyPer7 mRNA for 4, 2, and 1 h. 30 min after the last transfection, cells were treated with H_2_O_2_ for 30 min. At least 10,000 singlets were collected for all flow cytometry experiments. Data was analyzed using FlowJo 10.10.0 (BD). Violin plots were generated using R 4.4.0. Bar graphs were generated in Microsoft Excel.

## Supplementary Information

Below is the link to the electronic supplementary material.

Supplementary Material 1

## Data Availability

All data and materials generated through this study are available from the corresponding author on reasonable request. The whole-plasmid-sequencing data generated and analyzed in this study are available in the NCBI Sequence Read Archive (SRA) repository under BioProject accession number PRJNA1422863. In-vitro transcription templates generated in this study are available on Addgene (#255257-#255263).
