# Supplementary material for: Messenger RNA-encoded reporters for monitoring cellular stress and bioenergetics
Source: Sci Rep. 2026 Jun 17;16:18698. doi: 10.1038/s41598-026-49851-y (PMC13276251; doi:10.1038/s41598-026-49851-y)
Supplement: Supplementary file 1 — Supplementary Material 1 [file 41598_2026_49851_MOESM1_ESM.docx]

**Supplementary Information**

**Supplementary Figure S1. Validation of spectral independence for multi-organelle mRNA delivery.**

Pseudo-coloured confocal microscopy images of mRNA-encoded fluorescent proteins targeting the endoplasmic reticulum (moxGFP), mitochondria (mCherry), and Golgi apparatus (TagBFP), delivered simultaneously (top row) or individually (bottom three rows). Scale bars indicate 10 µm.

**Supplementary Figure S2. Evaluation of mRNA reporter dynamics and stability.**

1. Time-course expression of HyPer7 in U2OS cells. B510 and V510 expression of HyPer7 1, 2, and 4h after transfection, both with and without H_2_O_2_.
2. Flow cytometry analysis of HyPer7 expression in 201B7 (human induced pluripotent stem cells) after 1, 5, or 10 defrosts. mRNA was stocked at 1 µg/mL. 250 ng of mRNA was transfected into cells after each set of defrosts using Lipofectamine MessengerMAX.
3. Assessment of HyPer7 mRNA stability after 1, 5, or 10 defrosts using TapeStation. Lanes were loaded with either 100 ng (lanes 1-3) or 200 ng (lanes 4-6) of mRNA.
